# Supplementary material for: Nanofibrillar Green Composites of Polylactide/Polyhydroxyalkanoate Produced in Situ Due to Shear Induced Crystallization
Source: Polymers (Basel). 2019 Nov 4;11(11):1811. doi: 10.3390/polym11111811 (PMC6918183; doi:10.3390/polym11111811)
Supplement: Supplementary file 1 [file polymers-11-01811-s001.pdf]

Supplementary materials

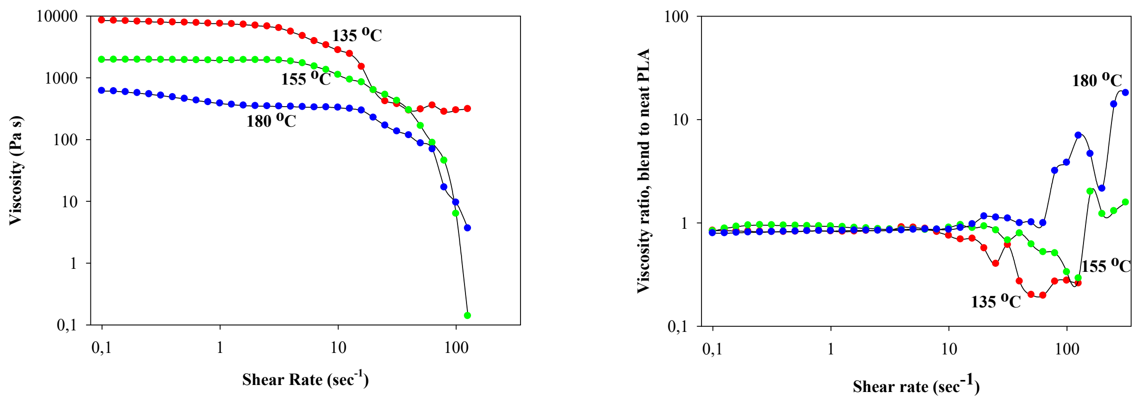

Fig. 1 a) Shear rate dependences of the complex viscosity for the matrix, PLA b) viscosity ratio of blend to matrix measured at 135, 155, 180 ˚C.

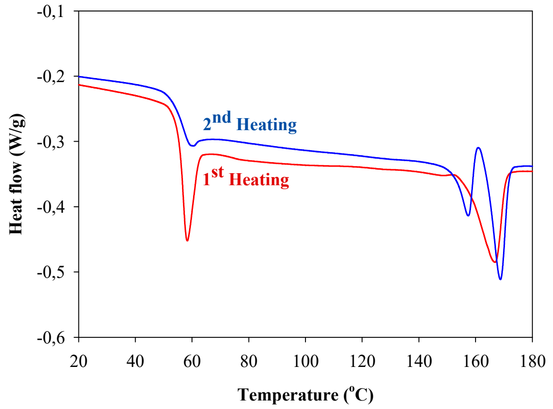

Fig. 2 Melting endotherm of PLA/PHA composite.

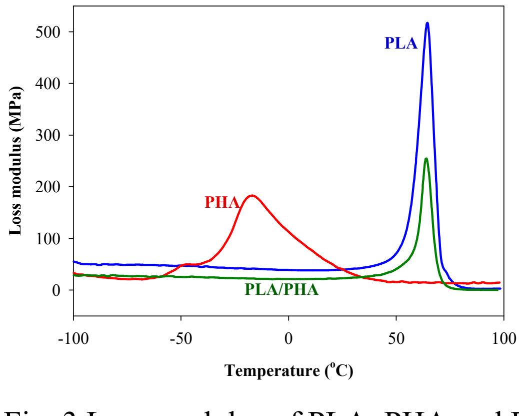

Fig. 3 Loss modulus of PLA, PHA and PLA/PHA composite in a tensile mode.

Table 1 Mechanical properties of PLA, PLA/PHA blend and composite

Sample	Young modulus, GPa	Yield stress, MPa	Stress at break, MPa	Strain at break, %
PLA	2.04	brittle fracture	43	7.0
PLA/PHA, blend	2.14	57	55	7.4
PLA/PHA, composite	2.35	61	56	28.1

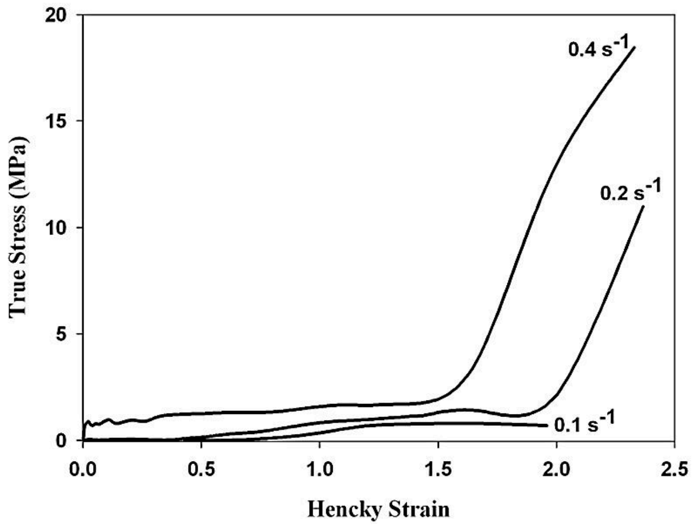

Fig. 4 True stress–Hencky strain curves of PHA nanofibers network for PLA/PHA nanocomposite determined at 100 °C and Hencky strain rates of 0.1, 0.2, and 0.4 sec^{-1}. Plots obtained by subtracting stress-elongation curve for neat PLA from respective stress-elongation curves for the nanocomposite.
